# Berberine and aspirin prevent traumatic heterotopic ossification by inhibition of BMP signalling pathway and osteogenic differentiation

**DOI:** 10.1111/jcmm.17919

**Published:** 2023-08-22

**Authors:** Jingjing Fan, Jiayu Gao, Jie Chen, Jia Hou, Mengchao Liu, Yanmiao Dang, Hui Lin

**Affiliations:** ^1^ Jiangxi Province Key Laboratory of Tumor Pathogenesis and Molecular Pathology, Department of Pathophysiology, School of Basic Medical Sciences Nanchang University Nanchang China

**Keywords:** AMPK, berberine; aspirin, BMP signalling, heterotopic ossification

## Abstract

Heterotopic ossification (HO) is a pathological process that often occurs in soft tissues following severe trauma. There is no effective therapy for HO. The BMP signalling pathway plays an essential role in the pathogenesis of HO. Our previous study showed that AMPK negatively regulates the BMP signalling pathway and osteogenic differentiation. The present study aims to study the effect of two AMPK activators berberine and aspirin on osteogenic differentiation and HO induced by traumatic injury. The effects of two AMPK activators, berberine and aspirin, on BMP signalling and osteogenic differentiation were measured by western blot, ALP and Alizarin red S staining in C3H10T1/2 cells. A mouse model with Achilles tenotomy was employed to assess the effects of berberine and aspirin on HO using μCT and histological analysis. First, our study showed that berberine and aspirin inhibited phosphorylation of Smad1/5 induced by BMP6 and the inhibition was attributed to the down‐regulation of ALK2 expression. Second, the combination of berberine and aspirin yielded more potent effects on BMP signalling. Third, we further found that there was an additive effect of berberine and aspirin combination on osteogenic differentiation. Finally, we found that berberine and aspirin blocked trauma‐induced ectopic bone formation in mice, which may be through suppression of phosphorylation of Smad1/5 in injured tissues. Collectively, these findings indicate that berberine and aspirin inhibit osteogenic differentiation in C3H10T1/2 cells and traumatic HO in mice, possibly through the down‐regulation of the BMP signalling pathway. Our study sheds a light on prevention and treatment of traumatic HO using AMPK pharmacological activators berberine and aspirin.

## 1 INTRODUCTION

Heterotopic ossification (HO) refers to the aberrant formation of ectopic bone in muscle and soft tissue, and often occurs following severe trauma, burns, amputation, hip replacements, etc.^1^ HO causes chronic pain, joint ankylosis and immobilisation.^2^, ^3^ Additionally, progressive HO in soft tissues has been found in one genetic disease called fibrodysplasia ossificans progressiva (FOP, OMIM#135100), which arises from activating mutations in *ALK2*. Patients start to develop symptoms in the first decade and dies approximately 56 years of age due to restriction of respiration.^4^, ^5^, ^6^, ^7^

Currently, prevention or treatment strategies for HO are limited and plagued by modest clinical outcomes. The suppression of HO mainly relies on nonsteroidal anti‐inflammatory drugs and perioperative radiation.^8^ Surgical excision was performed for the primary treatment of already formed HO, but the risk of surgery resection involves triggering the recurrence of HO.^9^ Therefore, a critical unmet need is developing therapeutic options for HO. Several signalling pathways are involved in HO, among which the bone morphogenetic proteins (BMPs) signalling pathway plays an essential role in the pathophysiology of HO formation.^10^, ^11^ BMPs belong to the transforming growth factor‐β (TGF‐β) family, regulating cells differentiation and bone formation.^2^ BMP signalling has fundamental roles in both embryonic skeletal development and postnatal bone homeostasis.^12^, ^13^ Dysregulated BMP signalling was associated with bone disorders in humans, including HO.^2^ BMP 2/4/9 promotes ectopic bone formation after being injected into the muscle tissue.^14^, ^15^ BMP/ALK2 signalling is over‐activated in the early stage of HO in many animal models.^16^, ^17^, ^18^ One clinical study revealed that patients with a traumatic brain injury have high serum level of BMP2.^19^ In keeping with these findings, by using the pharmacological inhibition of BMP/ALK2 signalling prevented trauma induced and genetic HO in animal models.^20^, ^21^, ^22^, ^23^, ^24^ Therefore, strategies to target the BMP/ALK2 signalling pathway have great potential to prevent HO formation.

AMP‐activated protein kinase (AMPK) is a fuel sensor kinase, which is activated in cells experiencing energy stress, such as ischemia and hypoxia.^25^ Upon activation, AMPK activates ATP generation catabolic processes and suppress anabolic processes in coping with metabolic stress. AMPK is an attractive treatment of the Type 2 diabetes, as it suppresses liver glucose production, reduces blood glucose and enhances muscle and adipose tissues glucose uptake.^25^, ^26^ Indeed, berberine and aspirin are well recognized pharmacological activators of AMPK.^27^, ^28^, ^29^ Berberine and aspirin activate AMPK through different mechanism. Berberine activates AMPK by inhibiting mitochondrial ATP synthesis and thus increase cellular AMP and ADP.^30^ Aspirin, a synthetic derivative, activates AMPK by binding at the ADaM (allosteric drug and metabolite) site as one allosteric activator and protection against Thr172 dephosphorylation.^29^, ^31^

Many studies have reported that AMPK is involved in osteogenesis.^32^, ^33^, ^34^, ^35^ Our previous studies have shown that AMPK activation inhibited osteogenic differentiation of induced pluripotent stem cells and MC3T3‐E1 cells and HO in mice.^34^, ^36^ The present study investigates the effect of berberine and aspirin on BMP signalling and the osteogenic differentiation of C3H10T1/2 cells, Furthermore, we establish a traumatic mouse model to evaluate the value of these two drugs in the prevention of HO formation. This study will be instrumental to develop their use in the clinical management of HO.

## 2 MATERIALS AND METHODS

### 2.1 Reagents

Recombinant mouse BMP‐6 protein was obtained from R&D System. Berberine, aspirin and, the BCIP/NBT Liquid Substrate System were obtained from Sigma‐Aldrich. The mesenchymal Stem Cell Osteogenic Differentiation Medium (MSCODM) kit and Alizarin Red S staining kit were obtained from Cyagen Biosciences. Anti‐total Smad1, phospho‐Smad1/5 (Ser463/465), total AMPKα and phospho‐AMPKα (T172) antibodies were obtained from Cell Signalling Technology, lnc. Anti‐β‐actin and Smad6 antibodies were obtained from Abcam. Anti‐Smurf1 and ALK2 antibodies were obtained from Santa Cruz Technology.

### 2.2 Cell culture and osteogenic differentiation

The C3H10T1/2 (murine mesenchymal stem cells) cells were purchased from the Cell Bank of the Chinese Academy of Sciences. The cells were cultured in DMEM containing 10% foetal bovine serum (FBS)and antibiotics. The cells were cultured in 5% CO_2_ at 37°C. Osteogenic differentiation of C3H10T1/2 was induced in osteogenic differentiation medium (MSCODM) according to the manufacturer's instruction.

### 2.3 Cell counting Kit‐8 assay

C3H10T1/2 cells were seeded into 96‐well plates (2000 cells/well). The cells were treated with berberine and then added 10 μL CCK‐8 solution (Yeasen Biotechnology). After 1 h, the OD value of each well was measured at 450 nm using a micro‐plate reader (SpectraMax Paradigm Multi‐Mode Detection Platform, Molecular Devices).

### 2.4 Alkaline phosphatase (ALP) staining and Alizarin red S staining

The C3H10T1/2 cells were cultured in osteogenic differentiation medium (MSCODM). The ALP staining was performed after 7 days using the BCIP/NBT Liquid Substrate System. Briefly, the cells were fixed and incubated with ALP staining solution for 1–2 h in the dark at room temperature. The cells were washed with distilled water and then the images were photographed under a light microscope. After 21 days, the C3H10T1/2 cells were fixed and stained with Alizarin red staining solution for 30 min for mineralisation. Cells were washed with distilled water and photographed. The positive stained cells were measured with the Image J software for quantification.

### 2.5 Traumatic HO Mouse Model

C57BL/6 female mice were obtained and housed under standard conditions in the Center of Laboratory Animal Science of Nanchang University. The animal protocols were approved by the Institutional Animal Care Committee of Nanchang University (NCDXSYDWFL‐2015097). The procedures were performed strictly in accordance with National Institutes of Health Guide for Animal Care. The mouse traumatic HO model was established by performing Achilles tenotomy and burn injury, which was reported in our previously study.^3^, ^34^ In brief, C57BL/6 female mice aged 6–8 weeks old underwent an Achilles tenotomy on the left hind limb under general anaesthesia. A metal (preheated to 60°C) block was placed on the mice dorsum for 20 s. For drug treatment, mice were treated with berberine (10 mg/kg) or aspirin (10 mg/kg) in DMSO or berberine plus aspirin or vehicle (DMSO) via intraperitoneal injection the day after burn/tenotomy. The mice received drugs or the vehicle for every 3 days (berberine, *n* = 4; aspirin, *n* = 4; vehicle, *n* = 4; berberine+aspirin, *n* = 4).

### 2.6 MicroCT analysis

The injured hind limbs were collected from each group (vehicle, *n* = 4; berberine, *n* = 4; aspirin, *n* = 4, berberine+aspirin, *n* = 4) at 8 weeks after burn/Achilles tenotomy, and were analysed with a MicroCT scanner (μCT 100, Scanco Medical AG) which was described in our previous study.^34^ The parameters were set as follows: a voltage of 70kv, a current of 200 μA, exposure time of 300 ms, a resolution of 14.5 μm per pixel, threshold = 200. Two‐dimensional images were captured and the three‐dimensional images were reconstructed. The HO volume was calculated by the Evaluation V6.5‐3 software.

### 2.7 Histologic analysis and Safranin O/Fast green staining

The hind limb from each mouse was collected after euthanization (vehicle, *n* = 4; berberine, *n* = 4; aspirin = 4; berberine+aspirin, *n* = 4) at 8 weeks post‐injury, The tissues were fixed in 4% phosphate‐buffered paraformaldehyde for 2 days and then were decalcified with 19% EDTA solution for 4–6 weeks. The tissues were embedded in paraffin. A series of 5 μm sections were processed to assess HO formation using haematoxylin and eosin staining and Safranin O/Fast green staining. The haematoxylin and eosin staining was conducted according to the manufacturer's manual (Solarbio). The Safranin O/Fast green staining was performed using the Modified Safranine O‐Fast Green FCF Cartilage Stain Kit (Solarbio) according to the manufacturer's instructions.

### 2.8 Immunohistochemistry

Five micrometres of paraffin sections were processed to deparaffinize and rehydrate in xylenes and different concentrations of ethanol. Antigen retrieval was performed with citrate solution pH 6. Sections were incubated with 3% hydrogen peroxide and blocked by 10% goat serum at room temperature for 30 min. The tissue sections were incubated with primary antibodies to pSmad1/5 overnight at 4°C. The tissue sections were probed with HRP conjugated secondary antibodies (PV‐6001 and PV‐6002, ZSGB‐bio) and then used DAB Peroxidase substrate (ZLI‐9017, ZSGB‐bio)to visualize, followed by counterstaining with haematoxylin. Images were acquired under a light microscope (OLYMPUS BX43, Olympus Corporation).

### 2.9 Western blot

Total proteins were extracted from injured tissue or cells using RIPA lysis buffer containing protease inhibitor cocktail. The extracts were centrifuged and the supernatant was collected. Equal amounts of proteins were loaded onto sodium dodecyl sulphate polyacrylamide gel electrophoresis (SDS‐PAGE) gel and then the proteins were transferred onto polyvinylidene difluoride membranes (Millipore Sigma). The membranes were blocked using 5% non‐fat milk and then incubated with indicated primary antibodies. The signals were developed by HRP‐conjugated secondary antibodies and were detected using the ECL kit.

### 2.10 Statistical analysis

All quantitative data were presented as the mean ± standard deviation (SD). *p* < 0.05 was set for significance differences, determined by Student's *t*‐test using GraphPad prism (8.0.2).

## 3 RESULTS

### 3.1 Berberine and aspirin inhibit the BMP signalling pathway in C3H10T1/2 cells

**FIGURE 1:**
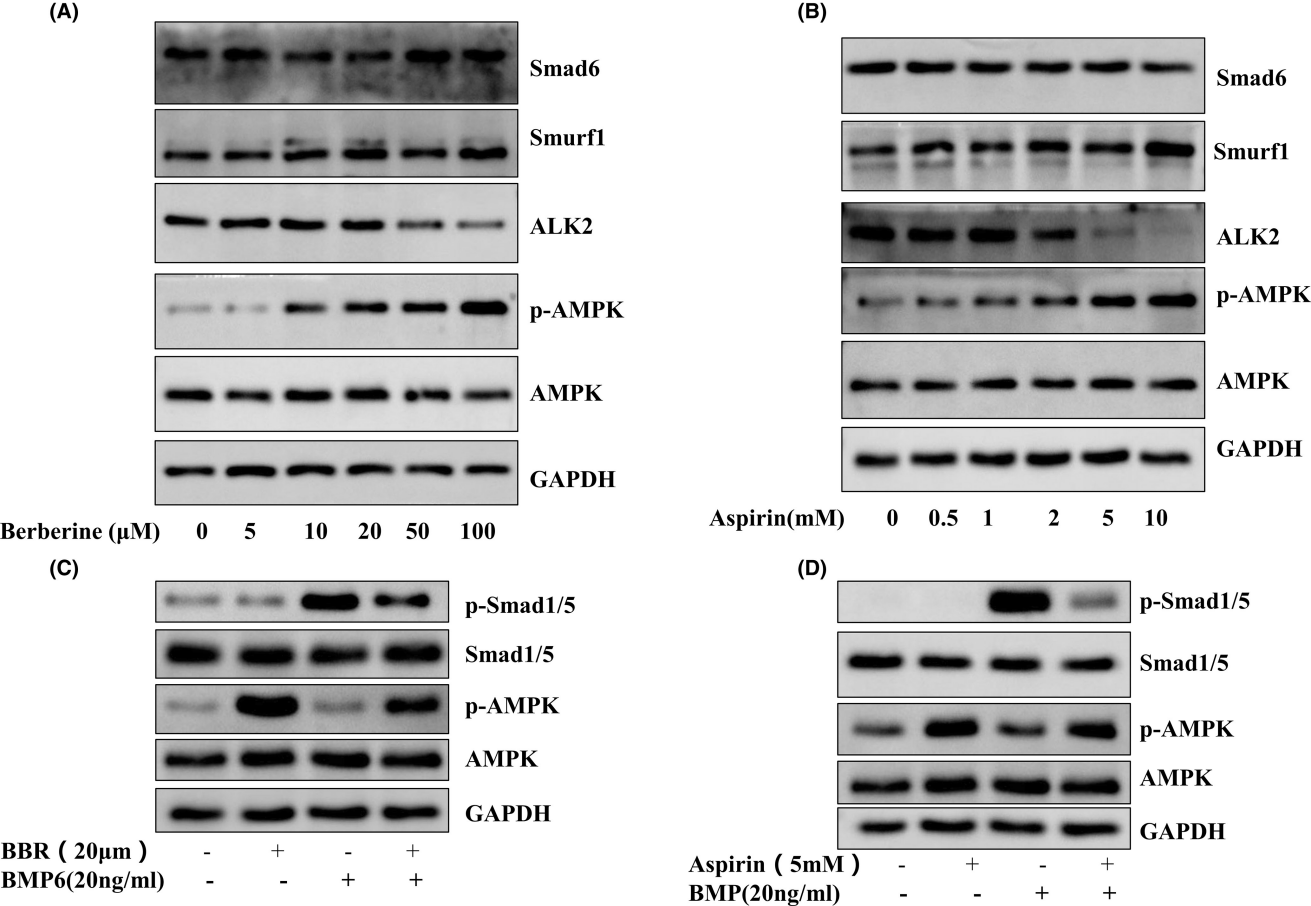
The effect of berberine and aspirin on the BMP signalling pathway. C3H10T1/2 cells were treated for 24 h with only berberine or aspirin with different doses (A, B), or followed by BMP6 (20 ng/mL) for 30 min (C, D). Western blot was performed with indicated antibodies. Each experiment was repeated three times independently and the representative blots were shown in the figure.

We first investigated the effect of berberine on C3H10T1/2 cells proliferation and cytotoxicity by CCK‐8 assay. We found that berberine (up to 100 μM) has no effect on cell proliferation activity for 48 h (Figure S1). We further studied the effects of berberine and aspirin on BMP signalling pathway in C3H10T1/2 cells. The C3H10T1/2 cells were treated with increasing doses of berberine (Figure 1A) and aspirin (Figure 1B) overnight. The data revealed that ALK2 expression was suppressed by berberine and aspirin in a dose‐dependent manner. The data also showed that the inhibition of ALK2 was in parallel with the degree of AMPK activation (Figure 1A,B). Interestingly, berberine and aspirin did not affect the protein expression of Smad6 and Smurf1.

Next, we examined if the reduction in ALK2 abundance concurred with its ability to phosphorylate Smad1/5. We treated the cells with berberine and aspirin overnight and then treated them with BMP6 for 30 min. As shown in Figure 1C,D, BMP6 significantly stimulated Smad1/5 phosphorylation, which was attenuated by berberine and aspirin treatment. A similar result was observed after density quantification (Figure S2). These results show that berberine and aspirin inhibit the BMP signalling pathway in C3H10T1/2 cells, which maybe mediated by ALK2 expression.

### 3.2 The additive effect of berberine and aspirin on BMP signalling pathway

**FIGURE 2:**
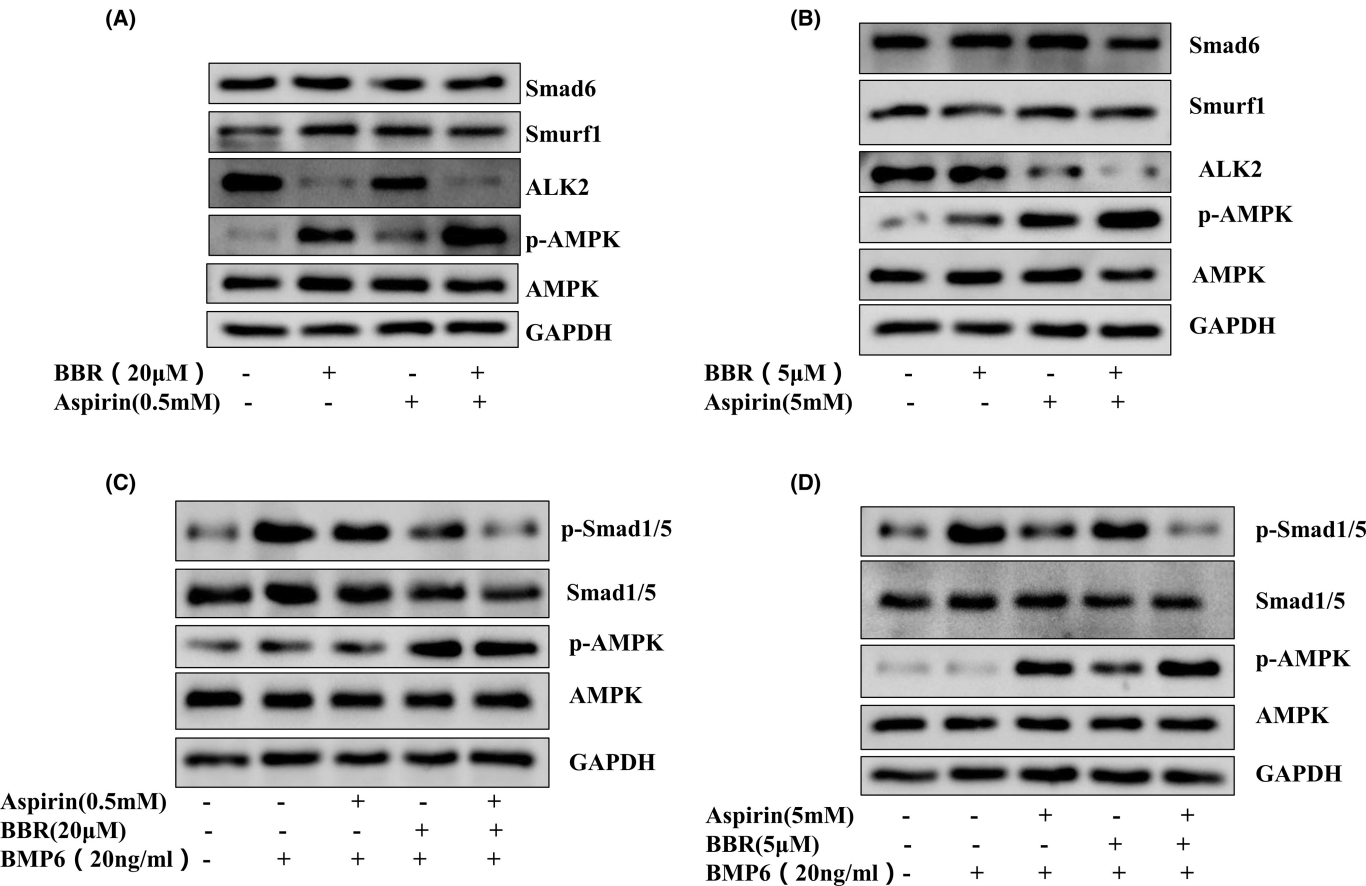
The combination effects of berberine and aspirin on the BMP signalling pathway. (A, B). C3H10T1/2 cells were treated with berberine and aspirin alone or in combination for 24 h. (C, D). The cells were treated with berberine and aspirin alone or in combination for 24 h, followed by BMP6 (20 ng/mL) for 30 min. Western blot was performed with indicated antibodies. Each experiment was repeated three times independently and the representative blots were shown in the figure.

We examined if an additive effect could be achieved when the two drugs were added together through different mechanisms of AMPK activation. Based on the Figures 1 and 2, we used 20 mM berberine (robust activation of AMPK) and 0.05 mM aspirin (weak activation of AMPK), or 5 mM berberine (weak activation of AMPK) and 5 mM aspirin (robust activation of AMPK) as combination. Our results reveal a further reduction of ALK2 protein levels in the combined treatment (Figure 2A,B), concurrent with the more potent activation of AMPK, as compared with a single treatment. Smad6 and Smurf1 have been shown negative regulation of BMP signalling pathway. We further study the effect of berberine and aspirin on Smad6 and Smurf1 expression. However, the expression of Smad6 and Smurf1 in C3H10T1/2 cells were unchanged after the combined administration of the two drugs. In conjunction, BMP6‐induced phosphorylation of Smad1/5 was proportionally suppressed by the combination of the two drugs (Figure 2C, D). A similar result was obtained after density quantification (Figure S3). These data show the additive effect of berberine and aspirin on the BMP signalling pathway after combination treatment in C3H10T1/2 cells.

### 3.3 Berberine and aspirin inhibit osteogenic differentiation of C3H10T1/2 cells

**FIGURE 3:**
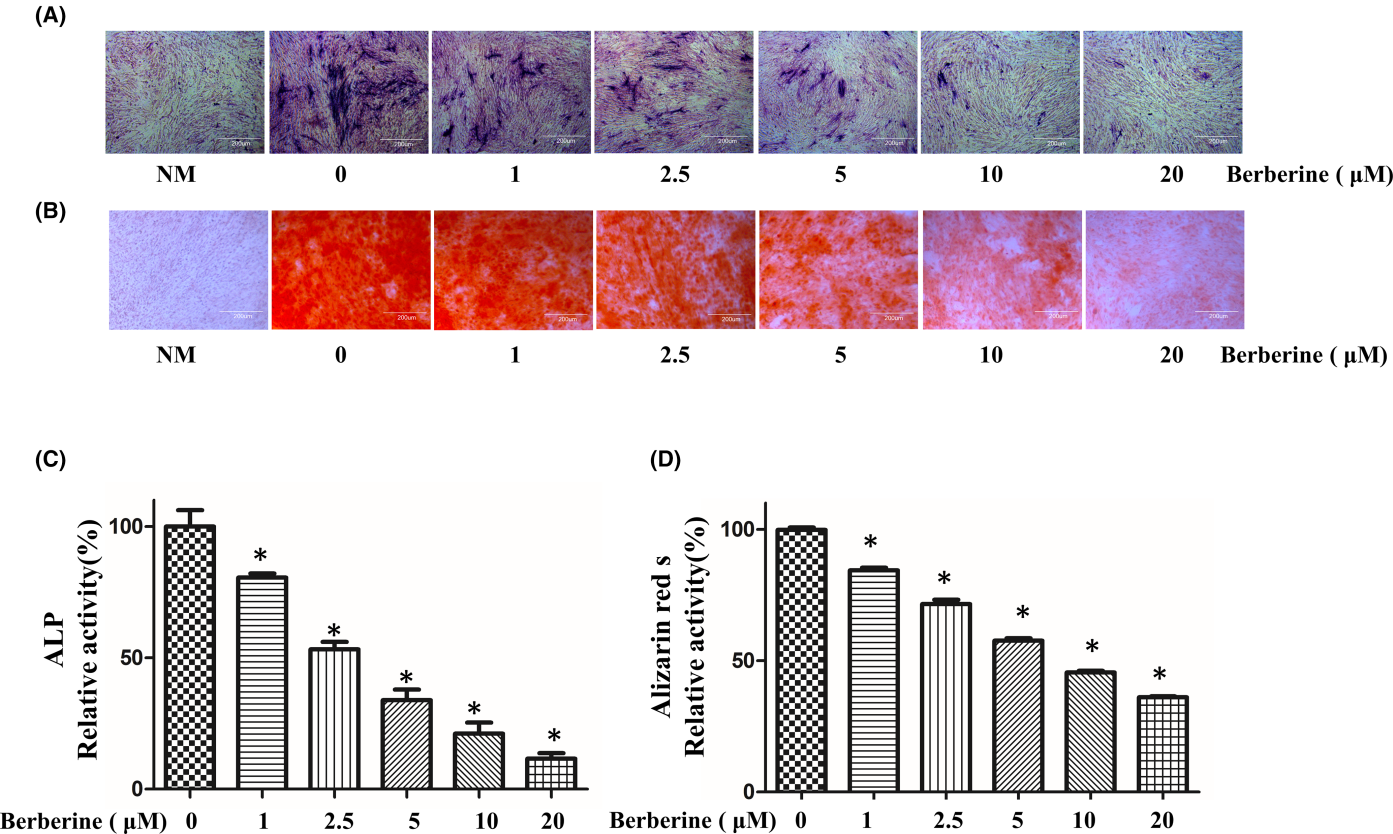
Effect of berberine on osteogenic differentiation of MC3T3‐E1 cells. (A, B). C3H10T1/2 cells were cultured in the MSCODM in the presence of different doses of berberine. ALP staining (A) and Alizarin Red S staining (B) were assessed after 7 days and 21 days, respectively. Representative images of ALP staining and Alizarin red s staining were shown in the figure. (C, D) Graph represents the stain intensity quantitation from three independent experiments (mean ± SD, *n* = 3) (C for ALP staining and D for Alizarin red staining). Significance was tested using Student's *t*‐test.*p < 0.05 as compared with control group. Scale bar = 200 μm.

Next, we investigated the effect of berberine and aspirin on the osteogenic differentiation of C3H10T1/2 cells. We first assayed the alkaline phosphatase (ALP, an early indicator of osteogenic differentiation) activity in C3H10T1/2 cells. The cells were cultured in MSCODM containing different doses of berberine and aspirin for 7 days. Berberine inhibited the ALP activity in a dose‐dependent manner (Figure 3A,C, Figure S4A).In addition; we assessed the effect of berberine and aspirin on the mineralisation (late stage indicator of osteogenic differentiation) of C3H10T1/2 cells. We found that berberine and aspirin progressively inhibited mineralisation with increasing doses (Figure 3B,D; Figure S4B). Our results demonstrate that berberine and aspirin suppresses the osteogenic differentiation of C3H10T1/2 cells.

### 3.4 Additive effect of berberine and aspirin on osteogenic differentiation of C3H10T1/2 cells

**FIGURE 4:**
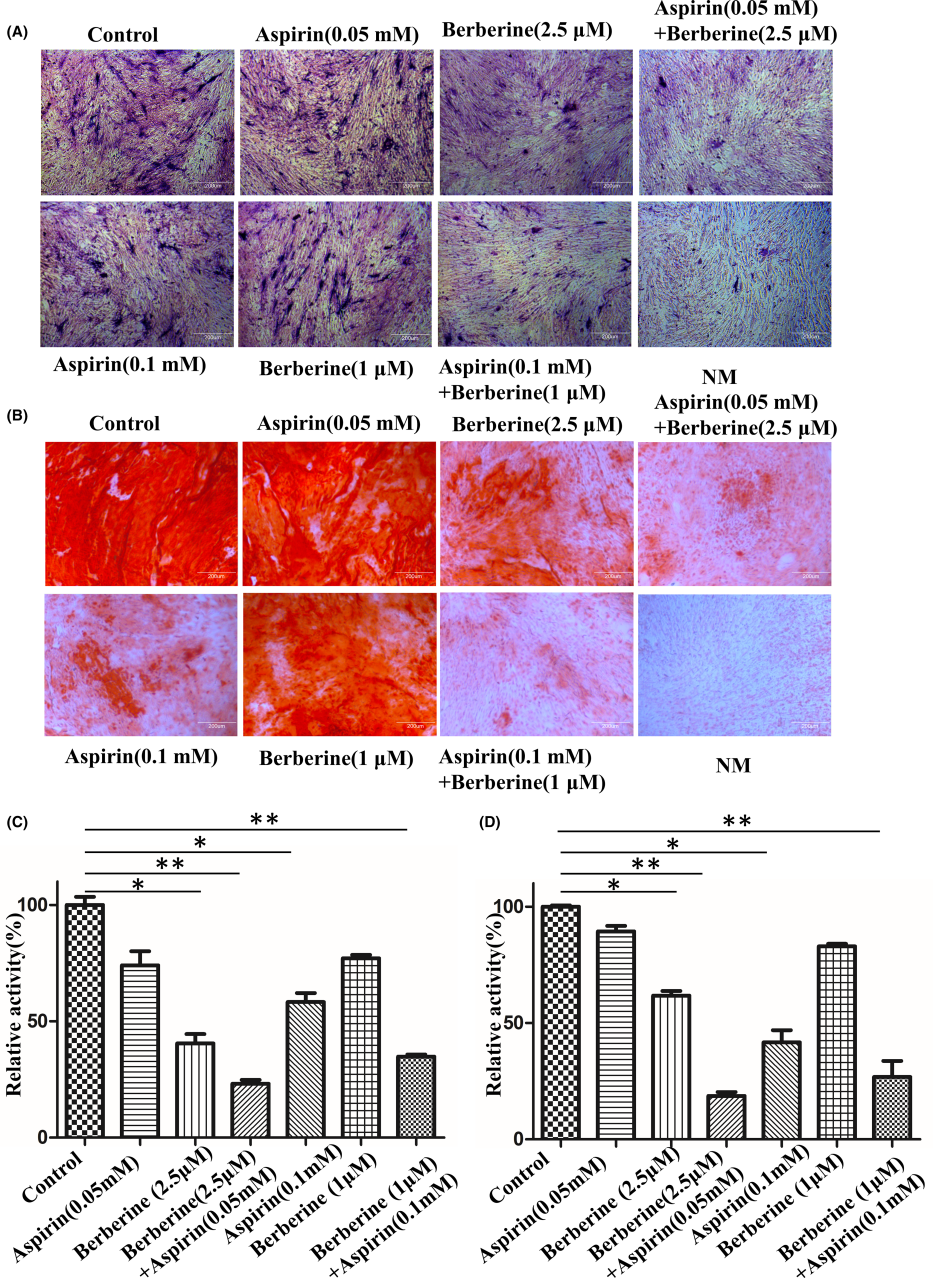
The Combination effect of berberine and aspirin on osteogenic differentiation. (A). C3H10T1/2 cells were cultured in the MSCODM and treated with berberine or aspirin or in combination at different doses. The culture medium was replenished every 3 days. ALP staining was performed after 7 days. (B). The differentiation of the cells was induced and treated as in (A). Alizarin red S staining was performed after 21 days. Representative images of ALP staining and Alizarin red S staining were shown in the figure. (C&D) Quantification of ALP (C) and Alizarin red S staining (D) were obtained from three independent experiments (mean ± SD, *n* = 3). NM: normal medium. Significance differences was tested using Student's *t*‐test with GraphPad Prism software.*p < 0.05 was set difference as compared with control group and indicated. Scale bar = 200 μm.

We further evaluated the effects of berberine and aspirin on osteogenic differentiation when the two drugs were added together. Based on previous data, we used 2.5 mM berberine (strong inhibition) and 0.05 mM aspirin (weak inhibition), or 1 mM berberine (weak inhibition) and 0.1 mM aspirin (strong inhibition) as combination. In the ALP activity assay (Figure 4A,C), compared with either berberine or aspirin alone, ALP activity were significantly inhibited by the combination of the two. Similar effects were observed on the mineralisation of C3H10T1/2 cells (Figure 4B,D). These results show that berberine and aspirin have additive effects on osteogenic differentiation of C3H10T1/2 cells.

### 3.5 Berberine and aspirin significantly reduced phospho‐Smad1/5 expression and trauma induced HO in mice

**FIGURE 5:**
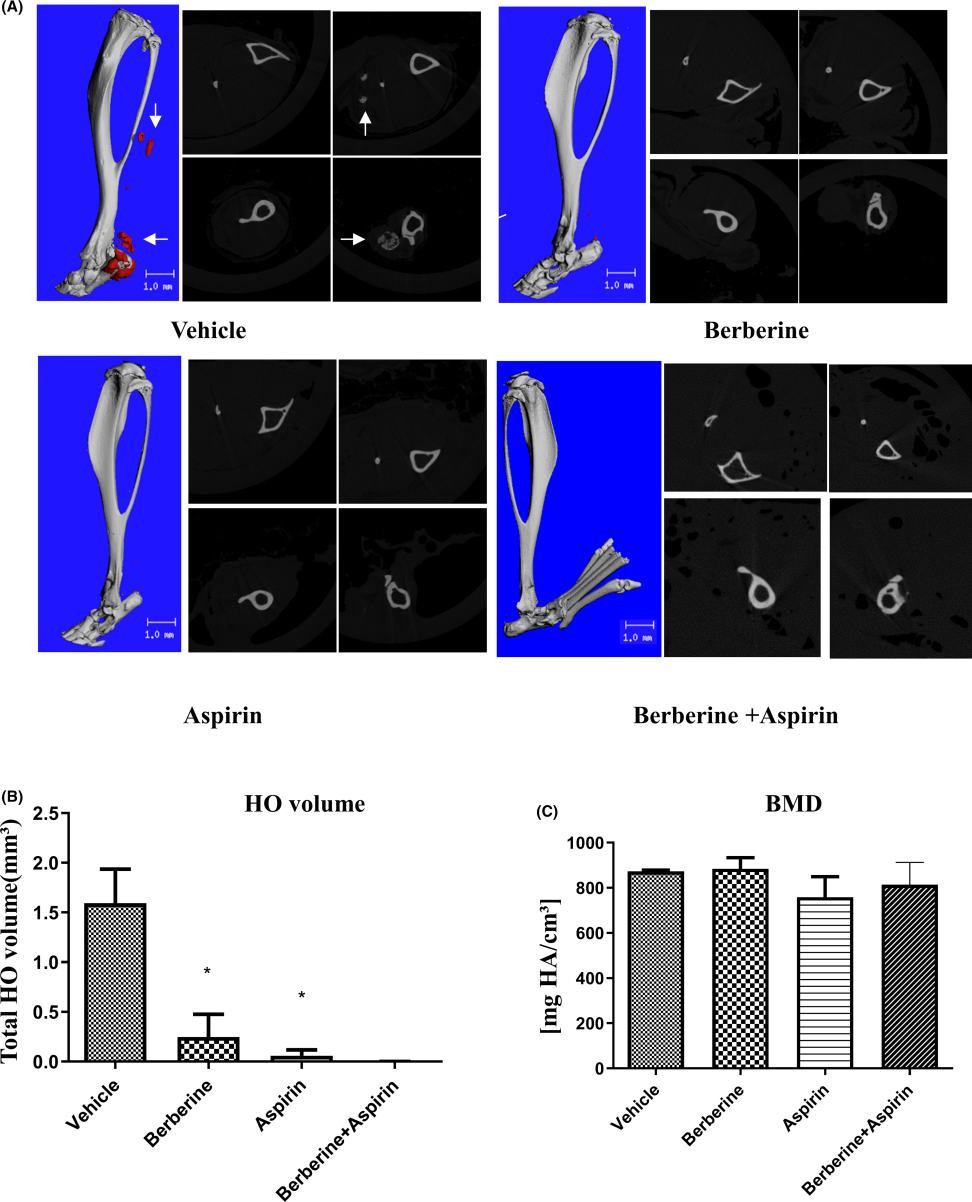
Berberine and aspirin prevent trauma‐induced HO in mice. (A). Mice received achilles tenotomy of the left hind limb and burn injury on the dorsum. The next day, mice were treated with berberine (10 mg/kg), aspirin (10 mg/kg), berberine plus aspirin and vehicle (DMSO) for 8 weeks(vehicle = 4; berberine, *n* = 4; aspirin, *n* = 4; berberine+aspirin, *n* = 4). The injured limbs were examined using Micro‐CT at 8 weeks post injury. Representative 3D microCT reconstructions and serial cross‐sections were shown in the figure. Scale bar = 1 mM. White arrows indicate heterotopic ossifications. (B, C).The volume (B) and bone mineral density(C) of HO were quantified from each group (vehicle, *n* = 4; berberine, *n* = 4; aspirin, *n* = 4, berberine+aspirin, *n* = 4). Significance was tested by Student's t test. *p < 0.05 as compared with vehicle group.

**FIGURE 6:**
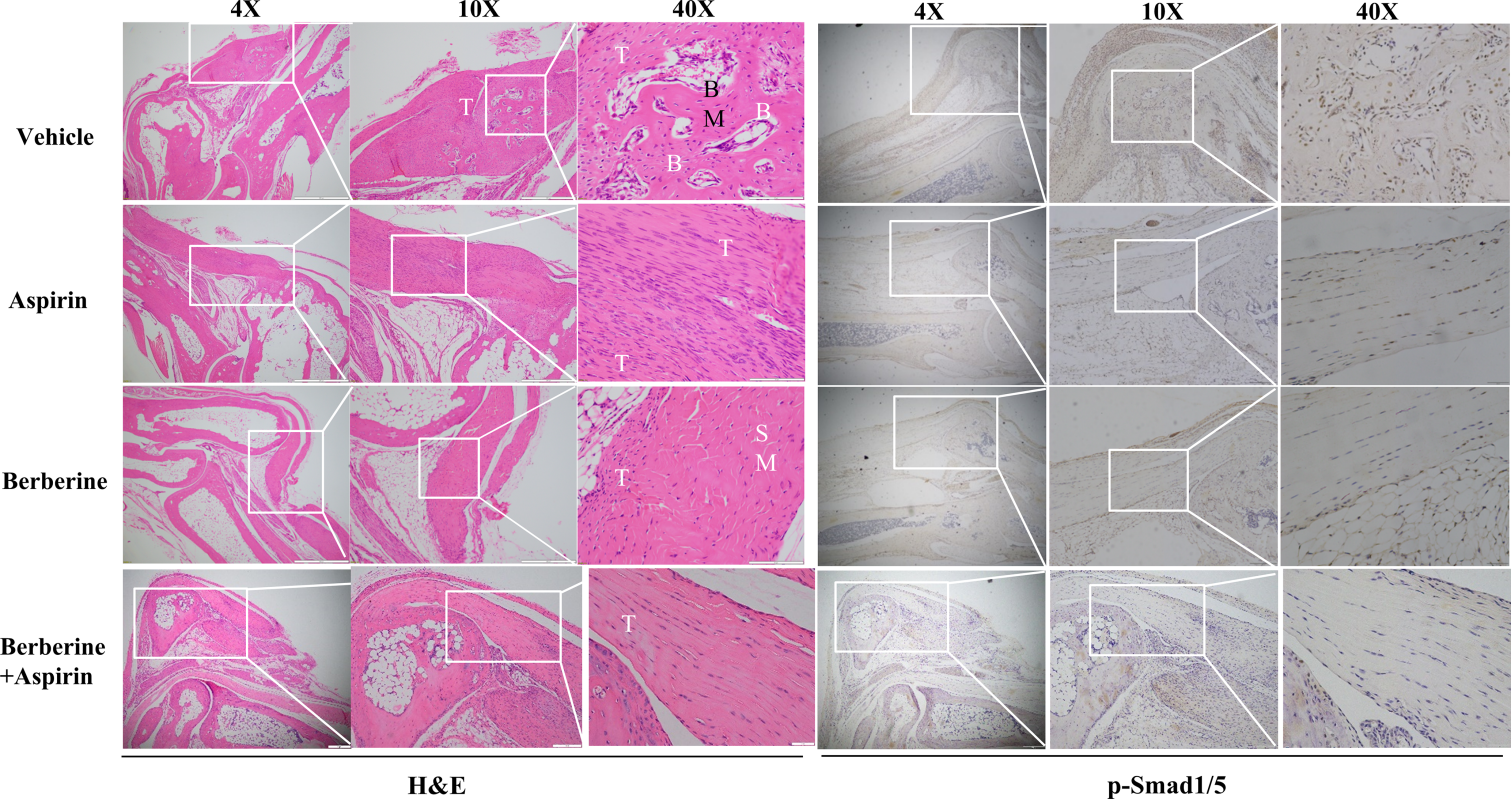
Haematoxylin and eosin staining was performed for heterotopic bone at 8 weeks after injury. (A) The injured tissues were collected from vehicle‐treated control, berberine or aspirin‐treated mice, and were sectioned and examined by haematoxylin and eosin staining (vehicle, *n* = 4; berberine, *n* = 4; aspirin, *n* = 4; berberine+aspirin, *n* = 4). (B) The injured tissues were collected from vehicle‐treated control, berberine‐treated or aspirin‐treated or berberine plus aspirin treated mice, sectioned and stained with p‐Smad1/5. Areas of interest in sections were examined at different magnifications, and the scales of 4X, 10X and 40X being 200, 100 and 20 μm, respectively. The representative images and the scale bar are shown in the figure. White arrow shows areas of interest. T: tendon, B: bone, SM: skeletal muscle.

Given that the dysregulated BMP signalling pathway participates in the pathogenesis of HO, the BMP/ALK2 signalling pathway was suppressed by berberine and aspirin. We further investigated the effects of berberine and aspirin on HO formation. Thus, we created a trauma‐induced HO mouse model by conducting Achilles tenotomy, followed by burn injury. Mice were treated with berberine (10 mg/kg, i.p.) or aspirin (10 mg/kg, i.p.) or berberine plus aspirin every 3 days after surgery. Micro‐CT scanning and reconstruction data demonstrated a significant reduction in HO formation in mice treated with berberine or aspirin for 8 weeks and volumes were calculated as well, the combination of berberine and aspirin showed more inhibitory effect in comparison to berberine or aspirin treated mice (Figure 5A,B).Interestingly, the density of ectopic bone was unchanged after berberine and aspirin treated (Figure 5C). Histological evaluation and Safranin O/Fast green staining confirmed the decrease of ectopic bone formation after berberine and aspirin treatment in mice (Figure 6A and Figure S5). Furthermore, immunohistochemistry staining revealed that berberine and aspirin‐treated mice showed down‐regulation in phospho‐Smad1/5 expression in injury sites in comparison with vehicle mice (Figure 6B), the combination treatment showed further suppressed the expression of phospho‐Smad1/5 expression in injury sites. Collectively, this study suggests that treatment with berberine or aspirin after injury reduce HO formation in mice, which may be through inhibition of BMP signalling pathway.

## 4 DISCUSSION

**FIGURE 7:**
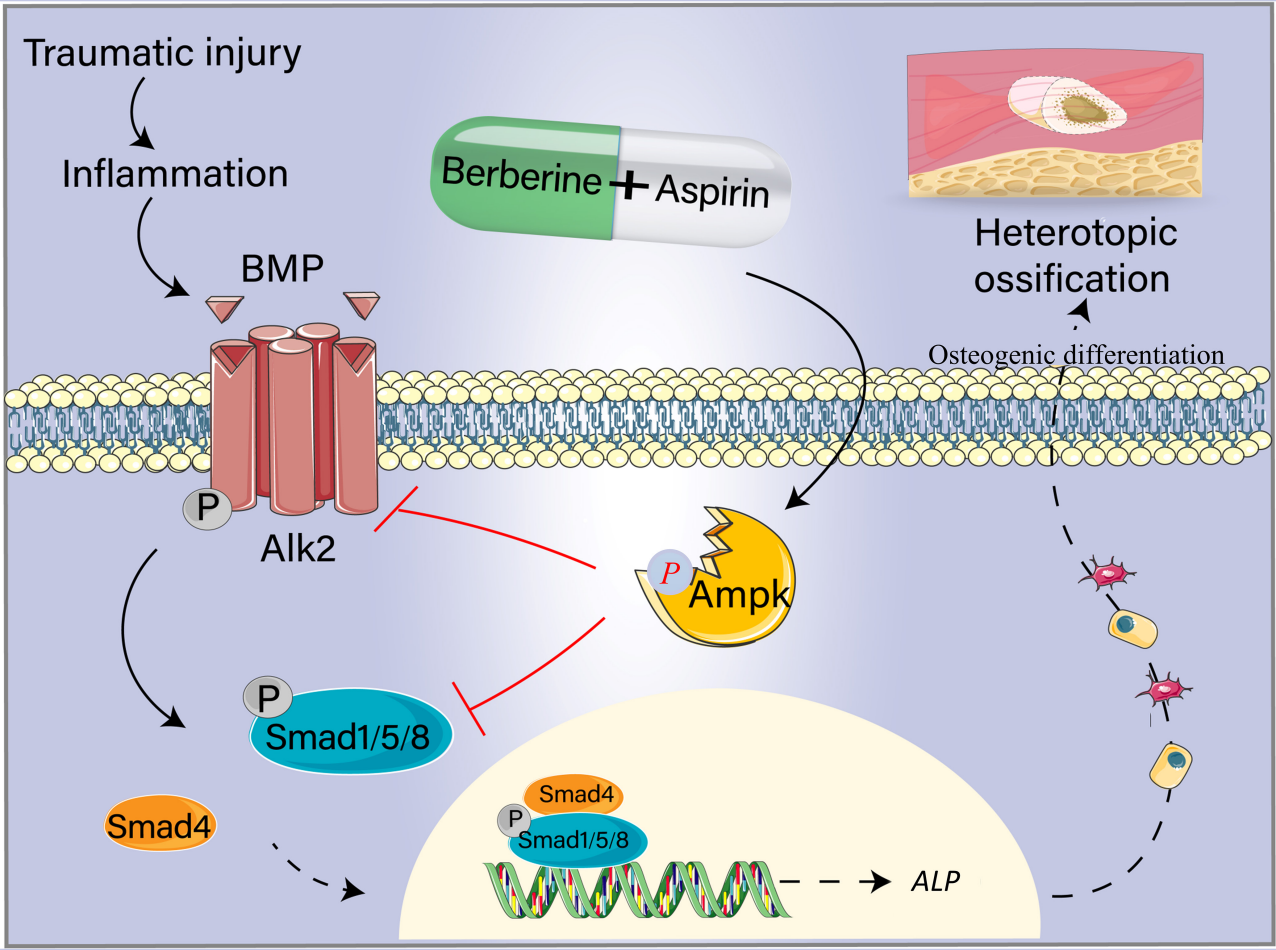
A working model of how berberine and aspirin prevent trauma induced HO. Berberine and aspirin could be potential anti‐HO drugs through the inhibition of the BMP signalling pathway and osteogenic differentiation.

HO is an aberrant formation of ectopic bone in soft tissues and muscles, a common complication occurs after severe trauma, amputations and hip replacements.^1^, ^2^, ^37^ Patients with trauma‐induced HO suffer pain, restriction of mobility and decrease in life quality. Currently, there are no effective treatment approaches for formed HO except for surgical resection. The pharmacological interventions are limited to non‐steroid anti‐inflammatory drugs that are used for the management of inflammation and symptomatic pain.^1^, ^38^, ^39^, ^40^ Thus, it is imperative to identify new therapeutic targets. BMP signalling plays important roles in both physiological and pathological osteogenesis. BMP signalling pathway is over‐activated by trauma injury and leads to HO.^41^ As such, blockers for BMP/ALK2‐mediated signalling could be used as therapeutic and/preventive drugs for HO. In this study, we investigated whether berberine and aspirin could have such a potential effect. We showed that both berberine and aspirin suppress BMP6‐induced phosphorylation of Smad1/5 in C3H10T1/2 cells. Second, we found that these two compounds decreased the osteogenic differentiation in vitro. Intriguingly, combination of berberine and aspirin generated additive effects on BMP signalling and osteogenic differentiation in parallel to the activation of AMPK. Finally, our data revealed that these drugs diminished phosphorylation of Smad1/5 expression and ectopic bone formation induced by traumatic injury in a mouse model. Collectively, our study provides us those pharmacological activators of AMPK could have value for HO therapy through the regulation of the BMP/ALK2 signalling pathway and osteogenic differentiation (Figure 7).

Efforts have been made to block the BMP/ALK signalling pathway to prevent HO formation. Thus, studies showed that selective small molecules inhibitors of Type I BMP receptors, have been used to reduce both trauma‐induced and genetic HO in animal model.^20^, ^22^ A3Fc, a BMP ligand trap of the ALK3 region that specifically binds to the ligand, has been used to reduce trauma induced HO.^22^ More importantly, an attractive approach is to identify drugs already approved for clinical use that could be repurposed for the management of HO. Palovarotene effectively prevented HO in transgenic mice carrying an ALK2 mutation and trauma‐induced mouse model.^42^, ^43^ By using a high‐throughput screening assay, dipyridamole, a powerful inhibitor of platelet aggregation, was identified as being able to prevent HO via acting on the BMP signalling pathway.^44^ Our study further finds berberine and aspirin block BMP signalling pathway and prevented HO formation. Moreover, for their combined use, the dosage of each in the pair can be adjusted if the side‐effect of one is intolerable for an individual.

Previous studies have shown that AMPK activity gradually decreased with the progression of osteogenic differentiation and that the pharmacological activation of AMPK blocks this process.^33^, ^34^ However, some studies have described that AMPK promotes osteogenic differentiation.^45^, ^46^ The reason for the discrepancy is not clear, which might be attributed to cell context. Recently, we have shown that the osteogenic differentiation of FOP fibroblast cells derived iPS cells and MC3T3‐E1 cells was inhibited by AMPK activation.^34^, ^36^ The present study involved the C3H10T1/2 mesenchymal cell line and obtained the same conclusion. Furthermore, our in vitro data are consolidated by the HO animal model. Likewise, many studies have demonstrated that berberine promotes osteogenic differentiation^32^, ^47^ and inhibits osteoclast formation and osteoporosis.^32^, ^47^, ^48^, ^49^ All these findings suggest that berberine is favourable for bone formation, which sounds paradoxical to our results. One plausible explanation might be that different mechanisms account for the bone formation under physiological and pathological settings. Indeed, the pathogenesis of HO involves inflammation and BMP signalling, both of which are suppressed by berberine and aspirin, while under the physiological setting, bone development involves more complex signalling pathways, such as Wnt, PI3K and BMPs.^1^

In conclusion, our study has shown that berberine and aspirin inhibit BMP signalling pathway and the osteogenic differentiation of C3H10T1/2 cells in vitro. The inhibitory effect of berberine and aspirin maybe mediated by reduction of ALK2 expression. Furthermore, the combination treatment shows that additive effect on BMP signalling pathway and the osteogenic differentiation. Finally, the administration of berberine and aspirin following trauma inhibits phosphorylation of Smad1/5 expression and prevents HO development in mice and the combination of berberine and aspirin showed more inhibitory effect on HO formation. Therefore, the present study shows the effect and underlying mechanism of berberine and aspirin on traumatic HO and offers us a new opportunity to assess them for the management of HO.

## AUTHOR CONTRIBUTIONS

**Jingjing Fan:** Conceptualization (equal); data curation (equal); formal analysis (equal); methodology (equal); visualization (equal); writing – original draft (equal). **Jiayu Gao:** Conceptualization (equal); data curation (equal); formal analysis (equal); software (equal); validation (equal); visualization (equal). **Jie Chen:** Conceptualization (equal); data curation (equal); validation (equal). **Jia Hou:** Conceptualization (equal); data curation (equal); formal analysis (equal); investigation (equal); software (equal); validation (equal). **Yanmiao Dang:** Conceptualization (equal); data curation (equal). **Mengchao Liu:** Data curation (equal); formal analysis (equal); methodology (equal); validation (equal). **Hui Lin:** Conceptualization (equal); data curation (equal); formal analysis (equal); investigation (equal); software (equal); supervision (equal); validation (equal); visualization (equal); writing – original draft (equal); writing – review and editing (equal).

## FUNDING INFORMATION

This work was supported by the National Nature Science Foundation of China (31900852 to HL), and, Nature Science Foundation of Jiangxi Province of China (20224ACB206024 to HL).

## CONFLICT OF INTEREST STATEMENT

The authors have no competing interests.

## Supporting information


Figure S1.

## Data Availability

The data that support the findings of this study are available from the corresponding author upon reasonable request.
