# Supplementary material for: Berberine and aspirin prevent traumatic heterotopic ossification by inhibition of BMP signalling pathway and osteogenic differentiation
Source: J Cell Mol Med. 2023 Aug 22;27(22):3491–502. doi: 10.1111/jcmm.17919 (PMC10660630; doi:10.1111/jcmm.17919)
Supplement: Supplementary file 1 — Figure S1. [file JCMM-27-3491-s001.docx]

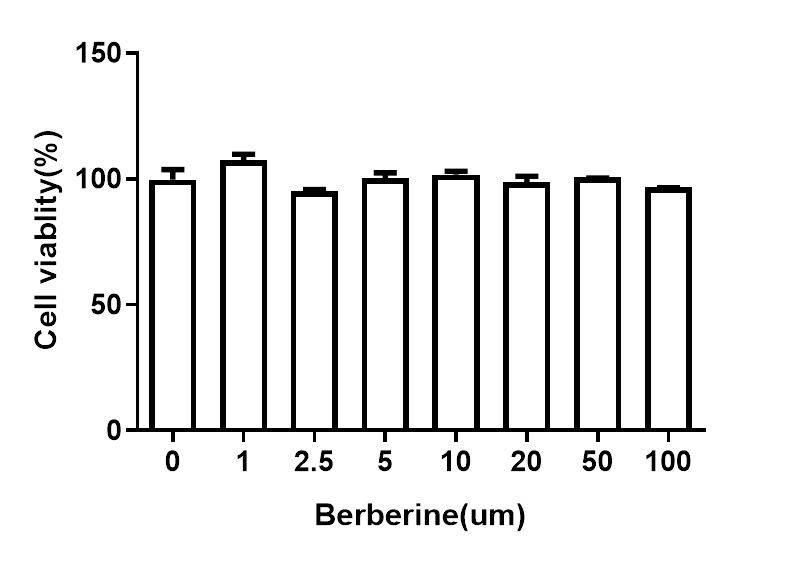


**Supplemental figure 1. The effect of Berberine on C3H10T1/2 cells proliferation activity by CCK-8 assay**

**
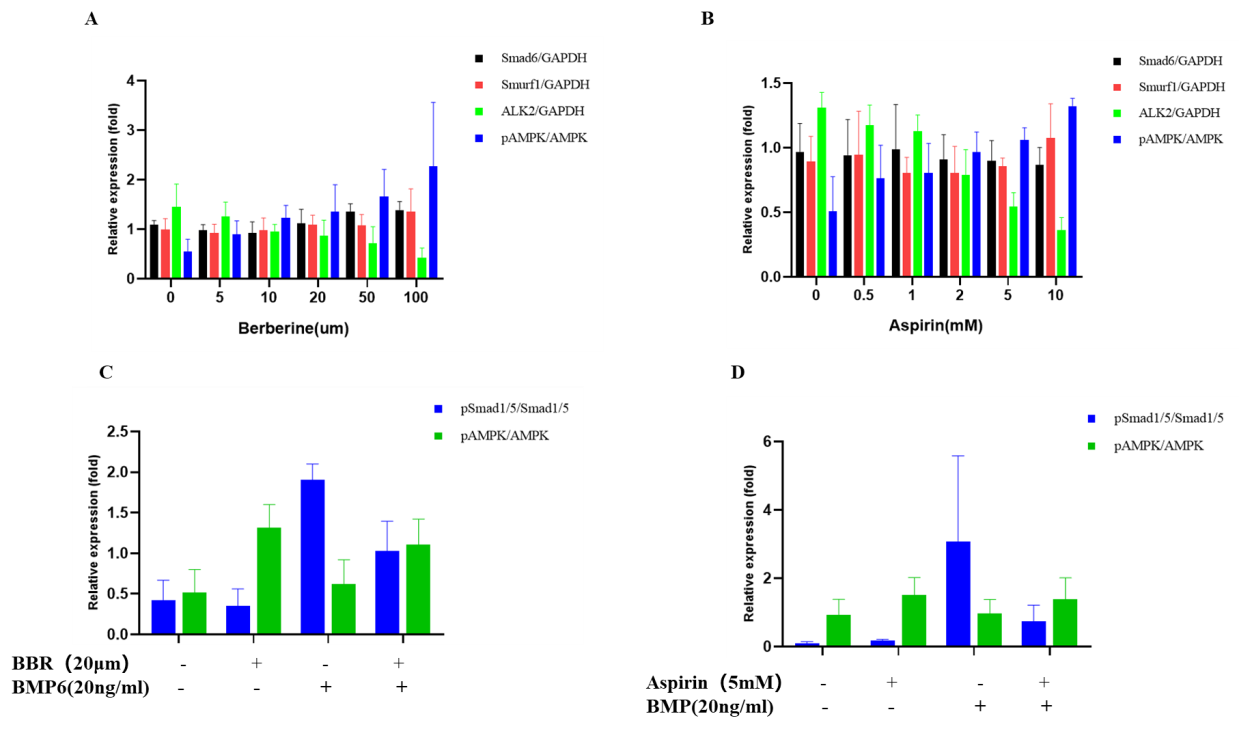
**

**Supplemental figure 2 .The effect of berberine and aspirin on the BMP signaling pathway**

C3H10T1/2 cells were treated for 24 fours with only berberine or aspirin with different doses (A&B), or followed by BMP6 (20ng/ml) for 30 min (C&D). Western blot was performed with antibodies, as indicated.  Graphs represent scan densitometric ratio of bands from three independent blots (Mean with SD).

**
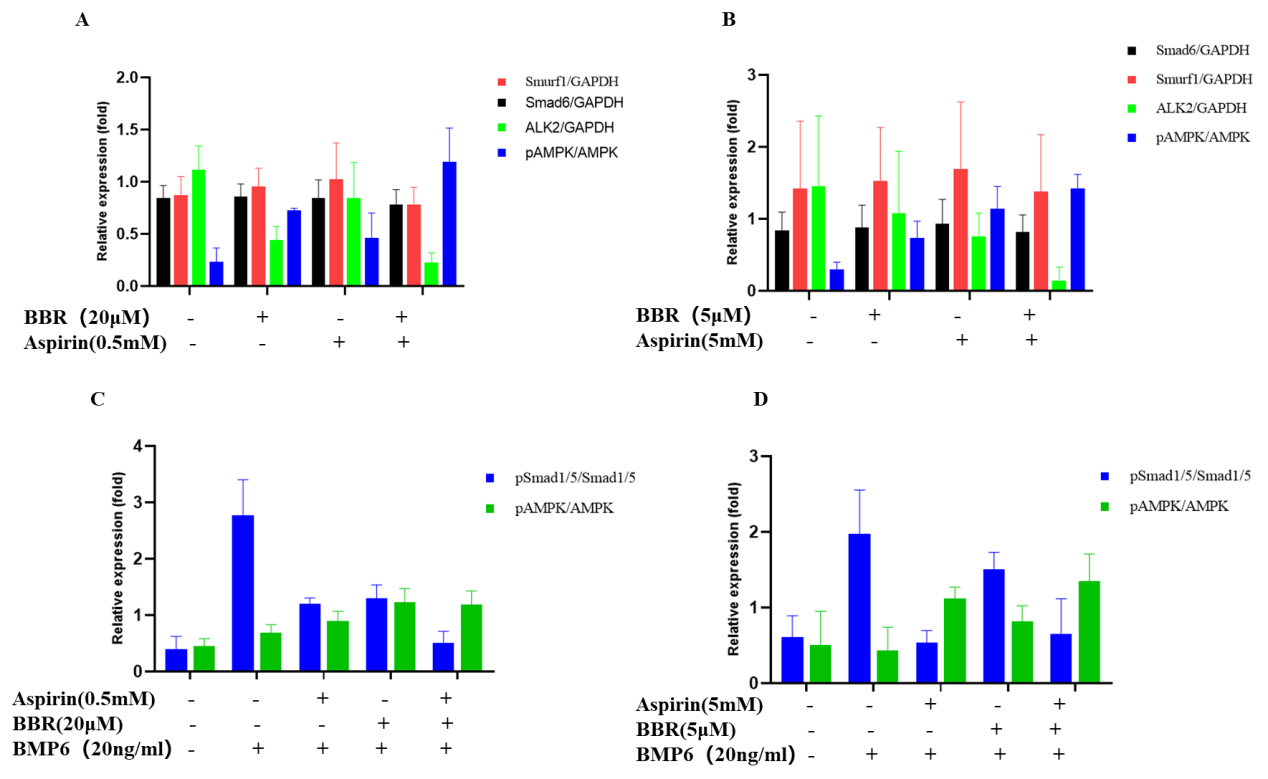
**

**Supplemental figure 3 .** The Combination effect of berberine and aspirin on BMP signaling pathway

A&B. C3H10T1/2 cells were treated with berberine and aspirin alone or in combination at different doses for 24 hours. C&D. The cells were treated with berberine and aspirin alone or in combination at different doses for 24 hours, followed by BMP6 (20ng/ml) for 30 min. Western blot was performed with antibodies, as indicated.  Graphs represent scan densitometric ratio of bands from three independent blots (Mean with SD).


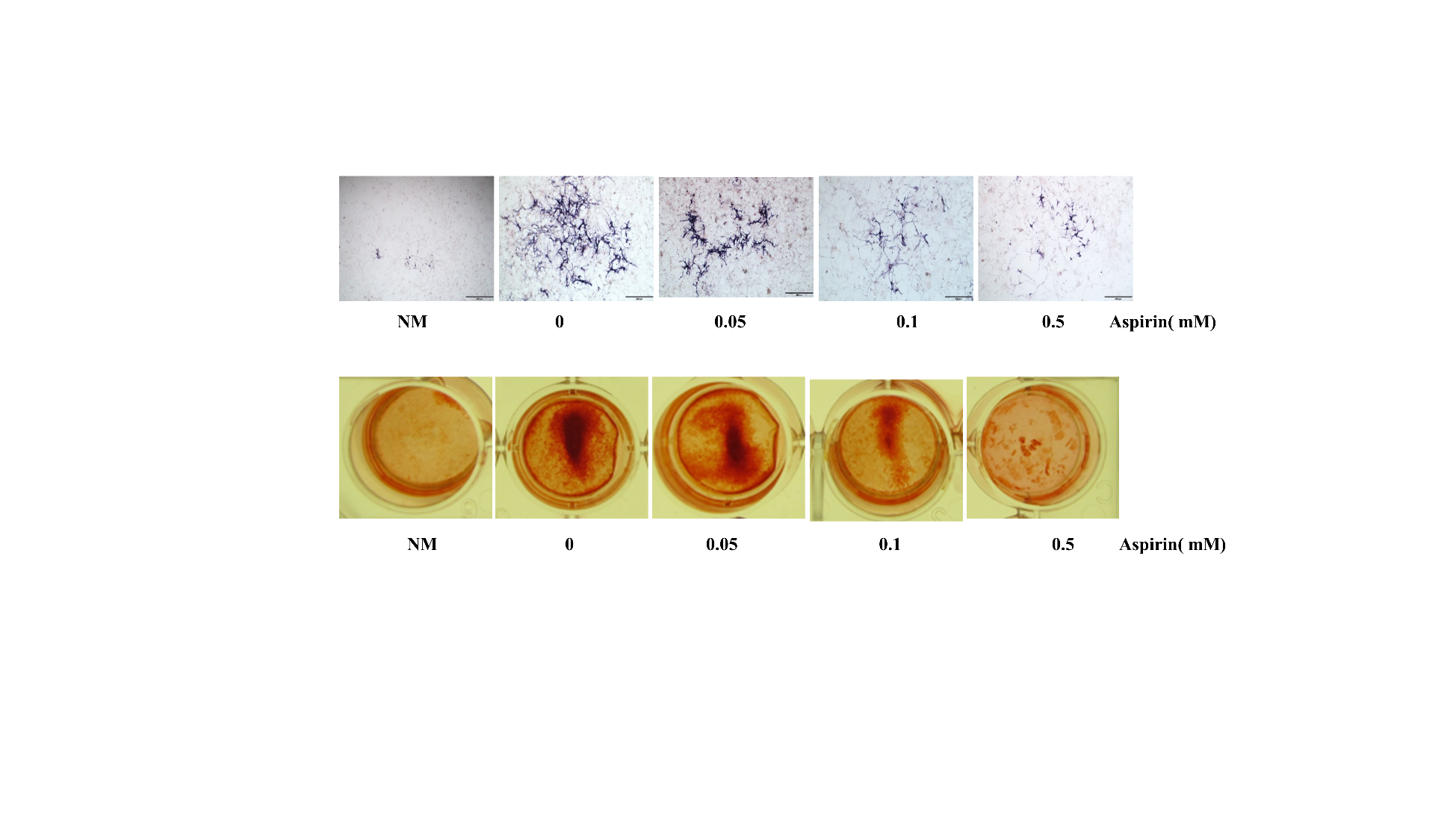


**Supplemental figure 4. Effect of aspirin on osteogenic differentiation of MC3T3-E1 cells**

A&B. C3H10T1/2 cells were cultured in the MSCODM in the presence of different doses of aspirin. ALP staining (A) and Alizarin Red S staining (B) were assessed after 7days and 21 days, respectively. Representative images of ALP staining and Alizarin red s staining were shown in the figure.


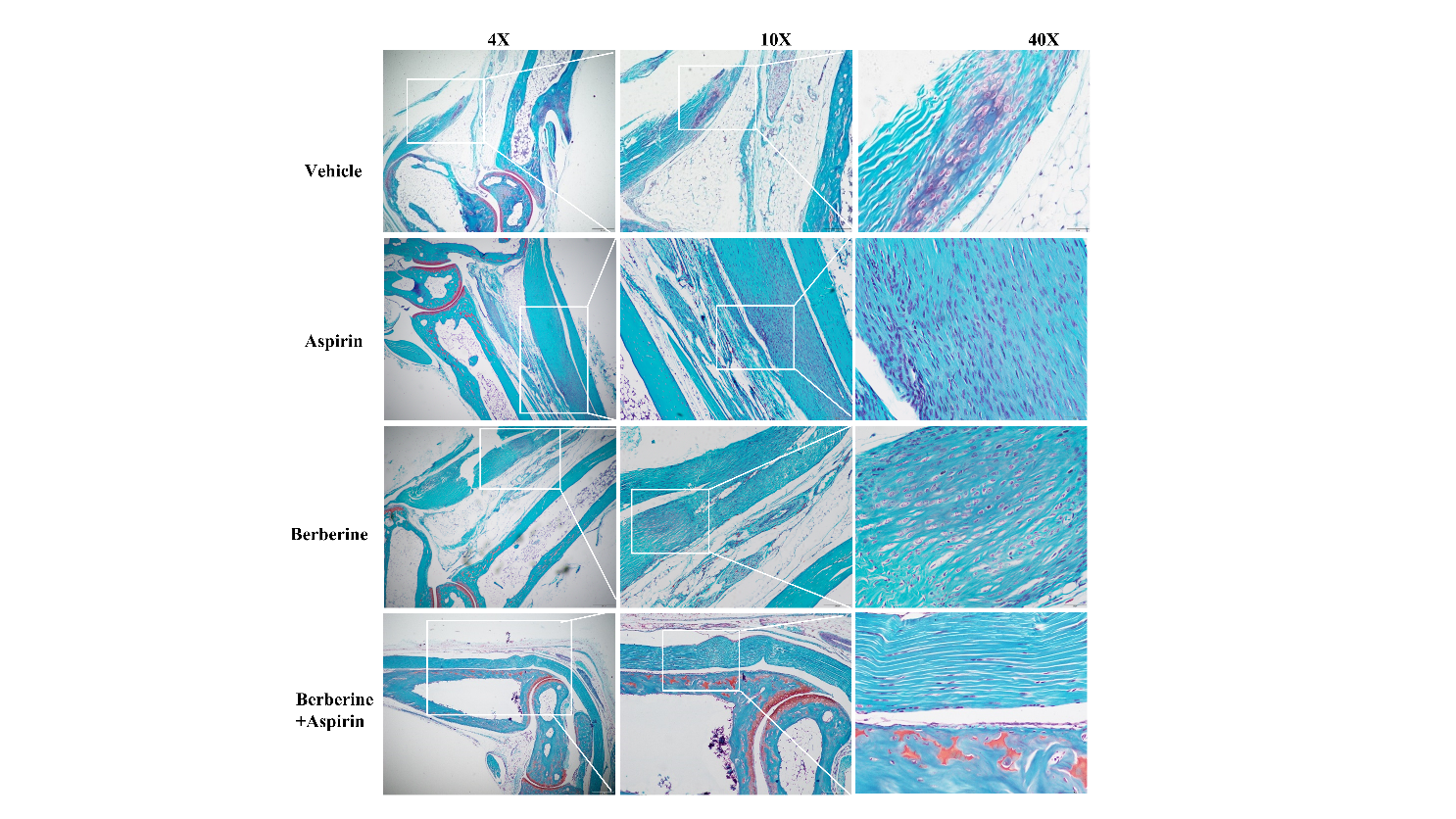


**Supplemental figure 5** . Mice received Achilles tenotomy of the left hind limb and burn injury on the dorsum. The next day mice treated with berberine(10mg/kg),aspirin(10mg/kg), berberine plus aspirin and vehicle (DMSO) for 8 weeks(vehicle=4,;berberine,n=4;aspirin,n=4; berberine+aspirin,n=4). The injured tissues were collected from vehicle-treated control, berberine or aspirin -treated or berberine plus aspirin treated mice, sectioned and examined by Safranin O/Fast green staining. Representative of Safranin O/Fast green staining of HO at 8 weeks after injury from each group. The scales of 4X, 10X, and 40X are 200um, 100um, and 20um, respectively.
